# Parylene C-Based Flexible Electronics for pH Monitoring Applications

**DOI:** 10.3390/s140711629

**Published:** 2014-07-01

**Authors:** Tatiana Trantidou, Mehvesh Tariq, Cesare M. Terracciano, Christofer Toumazou, Themistoklis Prodromakis

**Affiliations:** 1 Centre for Bio-Inspired Technology, Department of Electrical and Electronic Engineering, Imperial College London, London SW7 2AZ, UK; E-Mails: mehvesh.tariq10@imperial.ac.uk (M.T.); c.toumazou@imperial.ac.uk (C.T.); t.prodromakis@soton.ac.uk (T.P.); 2 National Heart and Lung Institute, Imperial College London, London W12 0NN, UK; E-Mail: c.terracciano@imperial.ac.uk; 3 Nano Research Group, Department of Electronics and Computer Science, University of Southampton, Southampton SO17 1BJ, UK

**Keywords:** Parylene C, flexible electronics, pH sensor, extended gate, discrete MOSFETs

## Abstract

Emerging materials in the field of implantable sensors should meet the needs for biocompatibility; transparency; flexibility and integrability. In this work; we present an integrated approach for implementing flexible bio-sensors based on thin Parylene C films that serve both as flexible support substrates and as active H^+^ sensing membranes within the same platform. Using standard micro-fabrication techniques; a miniaturized 40-electrode array was implemented on a 5 μm-thick Parylene C film. A thin capping film (1 μm) of Parylene on top of the array was plasma oxidized and served as the pH sensing membrane. The sensor was evaluated with the use of extended gate discrete MOSFETs to separate the chemistry from the electronics and prolong the lifetime of the sensor. The chemical sensing array spatially maps the local pH levels; providing a reliable and rapid-response (<5 s) system with a sensitivity of 23 mV/pH. Moreover; it preserves excellent encapsulation integrity and low chemical drifts (0.26–0.38 mV/min). The proposed approach is able to deliver hybrid flexible sensing platforms that will facilitate concurrent electrical and chemical recordings; with application in real-time physiological recordings of organs and tissues.

## 1. Introduction

Recent advances in the fields of material science and micro-fabrication technology have facilitated the implementation of flexible multifunctional electronics for *in vivo* bio-sensing applications. In contrast to intrinsically rigid sensing devices based on microelectrode arrays, such as the Utah array [1] or neural probes [2,3] flexible sensors are able to deliver sufficient spatial density and a minimally invasive and non-penetrating measurement interface. Furthermore, the high bendability and/or stretchability of these sensors make them ideal for tissue-attachable applications, as they can better adapt on skin and organ interfaces.

Research in the field of flexible sensors has mainly concentrated on “sophisticated skin” or “electronic skin” (e-skin), an electronic equivalent biological model that is realized by a large network of sensors on a stretchable substrate, usually a layer of polyimide (2–25 μm thick) for detecting pressure [4] or temperature [5]. Other studies have introduced innovative approaches in diagnostic devices for medical surgery of cardiac or brain therapy. Viventi *et al.* [6] employed a polyimide layer as the flexible structure substrate of a multilayered active circuitry, which was able to conformally attach on the epicardial tissue by soft contact and record the voltage data of cardiac activation from 228 measurement points. Recently, Rodgers *et al.* [7] implemented an inorganic electrode array on an ultrathin (2.5 μm) meshed polyimide substrate, demonstrating the ability of the sensor to make conformal contact on the brain of a cat and spatially map the physiological signals.

In all aforementioned implementations, polyimide has been chosen as the base substrate and insulating layer because of its high thermal and chemical resistance and mechanical robustness. Other studies have focused on other materials that are highly biocompatible, transparent and compatible with conventional micro-fabrication processes. For example, Rodgers *et al.* [8] fabricated high-density electrode arrays on stand-alone Parylene HT films (13.5–16 μm thick) for neural stimulation and recording in retinal and spinal cord prosthetics. Because of Parylene's chemical inertness, the material has been used more than thirty years now to suppress the pH sensitivity of ion-sensitive field effect transistors (ISFETs) and serve as a solid state reference electrode, namely reference field effect transistor (REFET), for differential ISFET/REFET pH measurements. The concept of the Parylene-gate FET (PGFET) has been introduced by Matsuo *et al.* [9], who deposited a thin (100 nm) film of Parylene on a Si_3_N_4_-gate ISFET to make it chemically inert. Nonetheless, the PGFET was pH sensitive, demonstrating a maximum sensitivity of 28 mV/pH below pH 4, whereas for larger pH values the sensor was ion insensitive. Chemical modification of Parylene's surface with crown ether compounds was also implemented to examine the K^+^ sensitivity of the REFET, nevertheless, problems such as decreasing ionic sensitivity response over time and large drift were reported. Fujihira and colleagues [10] attempted to enhance the encapsulation quality of Parylene N in chemically sensitive FETs (CHEMFETs) by experimenting with different cleaning procedures and chemical treatments of Si_3_N_4_ before Parylene deposition, and also through esterification of the Parylene's surface acids (mostly carboxyl and hydroxyl groups). They demonstrated a decrease of Parylene's pH sensitivity to 4 mV per decade pH within the range of pH 4–10 and even lower outside this range. Nonetheless, the stability of the Parylene REFET was highly subject to contamination and/or surface chemical reactions, which further compromised the accuracy of the ISFET/REFET system.

Other studies have exploited Parylene's high biocompatibility and waterproofness to conformally coat implantable microdevices, such as pacemakers [11], microelectrodes [12] and microelectromechanical devices for intraocular pressure monitoring [13]. Furthermore, thanks to its excellent insulating properties, Parylene was used to package Complementary Metal Oxide Semiconductor (CMOS)-based sensors in order to maintain leak signals at low levels [14,15]. The material's excellent encapsulation properties have been attributed to its pinhole-free nature above 1 μm, [16] but also to its hydrophobic surface that repulses any aqueous solution. In addition to its numerous applications, Parylene's mechanical robustness facilitates highly bendable ultrathin membranes down to 1 μm thickness that makes the material an ideal candidate for implantable sensors.

Previously, we have demonstrated that the H^+^ sensing capacity of Parylene C can be activated through plasma oxidation [16]. Here, we exploit this technology and present the application of Parylene C in flexible electronics in a smart-skin packaging. We demonstrate a versatile method for producing flexible Parylene C-based pH sensors which employ the material both as a flexible structural support and as a functional material to capture and transmit chemical inputs. The proposed work highlights a novel multifunctional approach for Parylene C, which can be employed in measuring multifunctional signals (electrical and chemical) in hybrid platforms.

## 2. Experimental Section

### 2.1. Sensor Fabrication

**Figure 1.**
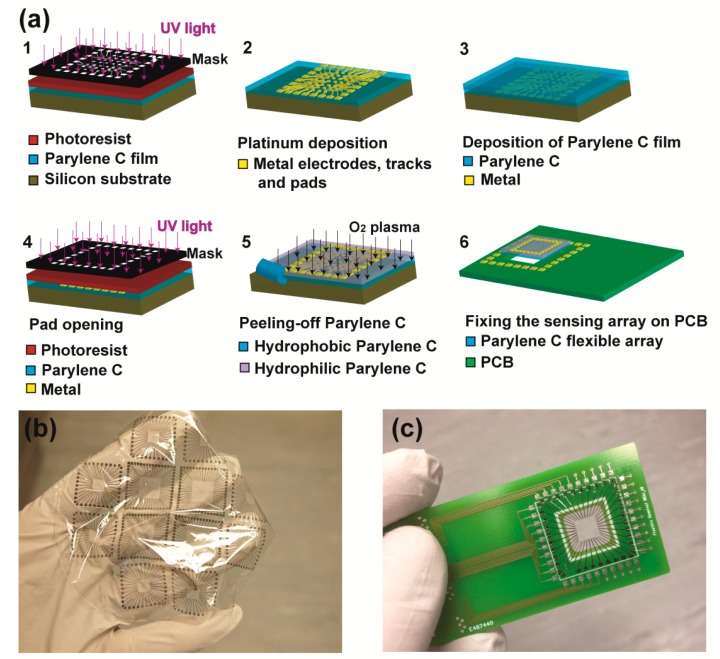
(**a**) Schematic of fabrication process of flexible sensor; (**b**) 5 μm-thick Parylene C flexible pH sensing arrays; (**c**) PCB-based prototype with the flexible chemical array located in the middle.

The fabrication process of the sensor is illustrated in Figure 1a. Four inch Si wafers were thoroughly cleaned with acetone (ACE), isopropanol (IPA) and deionised (DI) water, and blown dry with nitrogen. Five μm of Parylene C were deposited on the wafers through chemical vapor deposition (Labcoater PDS 2010, SCS, Indianapolis, IN, USA) to form the flexible support substrate. The wafers were spin-coated with a thin layer (1.5 μm) of photoresist (AZ5214E), soft-baked at 90 °C for 1 min, and then lithographically patterned. Electron gun deposition of Pt (10 nm) facilitated a 40-electrode array (Ø30 μm electrodes-200 μm spacing) on the Parylene surface. Subsequently, a 1 μm-thick Parylene film was deposited on top of the array to serve as the sensing membrane. Access to the underlying pads was accomplished through a second lithography using a thick layer of photoresist (7 μm of AZ4562, softbake at 100 °C for 1 min) and O_2_ plasma etching (Nano UHP, Diener electronic GmbH, Nagold, Germany, 400 W/31 min at a working pressure of 0.8 mbar). Finally, the H^+^ sensing capacity of the array was activated through plasma oxidation (400 W/10 min at a working pressure of 0.8 mbar), leaving a thin Parylene film (∼200 nm) on top of the electrode grid. The Parylene membrane was peeled-off (Figure 1b), fixed on a Printed Circuit Board (PCB)-based prototype with an epoxy resin and the pads were wire-bonded to the PCB (Figure 1c) to enable the monitoring of the chemical potentials.

Because the adhesion between the encapsulation layer and the underlying material plays an important role for the efficiency of the encapsulation, in our study we have chosen platinum to fabricate the miniaturized electrode array instead of other metals (e.g., gold) based on previous studies reporting that adhesion of Parylene C is significantly stronger on Pt substrates at similar deposition pressure (∼20 mTorr) [17]. Furthermore, it was also demonstrated that Parylene-to-Parylene adhesion was substantially high. In agreement to this observation, in our study we did not observe any adhesion issues, as the deposition of Parylene on top of an existing Parylene C layer formed a well-sealed “sandwiched” Parylene structure (Parylene-platinum-Parylene). However, to further improve the adhesion properties of Parylene, a pre-treatment of the surface with an adhesion promoter, for example Silane A-174, can be employed [17].

### 2.2. Read-Out Circuitry

**Figure 2.**
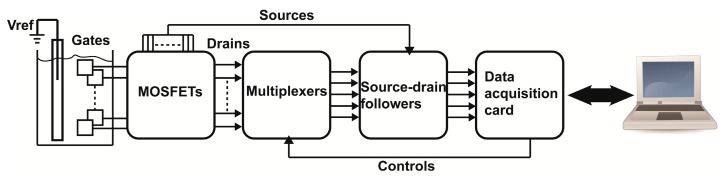
Overview of system architecture of the instrumentation used to evaluate the chemical sensors.

To evaluate the chemical performance of the sensing device, the PCB prototype was remotely connected to the gates of discrete p-type Metal Oxide Semiconductor FETs (MOSFETs) through an FFC/FPC connector. A universal instrumentation board was employed to host the inlet ports, the MOSFETs, a multiplexing scheme with the corresponding control pins, the appropriate biasing circuitry consisting of dual operational amplifiers source-drain followers and the outlet ports. Detailed description of the instrumentation board can be found in [18]. The control pins of the multiplexors and the drain outputs were controlled by corresponding software in MATLAB^®^ via a data acquisition card (NI-USB6210, National Instruments, Austin, TX, USA). A simple schematic showing the architecture of the read-out system is depicted in Figure 2.

The operational principle of these sensors is identical with ISFETs, where the threshold voltage of the transducer varies in relation to the electrolyte pH. Clearly, other circuitry can be used to deliver a more compact and responsive transducing system (e.g., instrumentation amplifiers). The extended gate protocol was employed in this study in order to separate the sensing part from the readout electronics and, therefore, increase the reliability and lifetime of the chemical sensors, which is a key aspect for all implantable sensing applications. Depending on the H^+^ concentration in the solution, the ions bind with the free bonds that are present on the plasma oxidized Parylene membrane. Binding of the H^+^ in the vicinity of an electrode introduces a local capacitance at the corresponding gate of the MOSFET, which leads to a corresponding output (drain) voltage. This in turn changes the threshold voltage of the MOSFET, allowing straightforward correlations between pH and output (drain) voltage.

### 2.3. Chemical Experiments

The chemical performance of the sensor was evaluated with 50 μL drops of phosphate buffer solutions of known pH (7004, 7007, 7010, HANNA Instruments, Leighton Buzzard, UK). A miniature Ag/AgCl reference electrode was used as a remote gate and was immersed in the microdroplet during measurements. Each electrolyte solution was applied to the sensor for 3 min for the sensitivity experiments and for 1 h for the drift experiments in the following order: pH 4, 7 and 10. Before testing a new electrolyte, the sensors and the reference electrode were rinsed with DI water to remove any residual chemical compounds and wiped carefully. For the hysteresis experiments, the sensor's output voltage was continuously recorded after applying electrolyte solutions in the following order: pH 10, 4, 7, 10, 7 and 4. All experiments were carried out at room temperature inside a faraday cage to minimize noise.

## 3. Results and Discussion

### 3.1. Chemical Sensitivity

**Figure 3.**
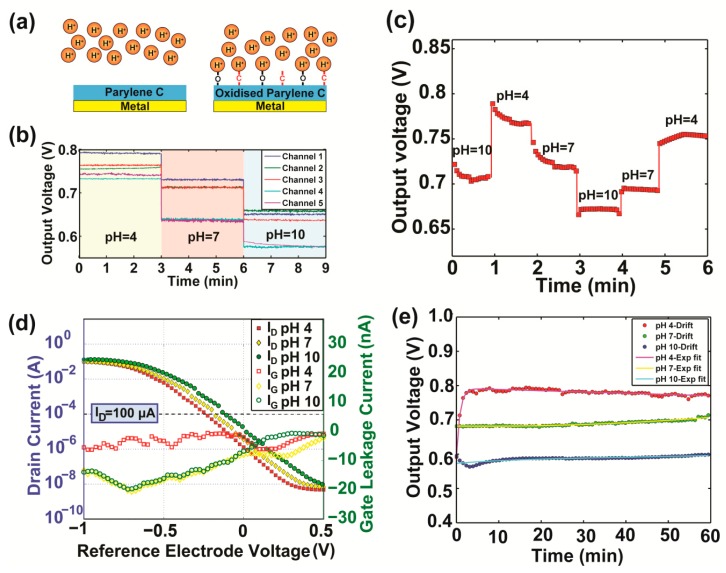
(**a**) Schematic diagram showing the sensing mechanism for untreated (hydrophobic) Parylene C (left) and oxidized Parylene C (right) sensing membranes; (**b**) Filtered data indicating chemical sensitivity of the sensor at distinct pH values; (**c**) Hysteresis characteristics (unfiltered data); (**d**) (Left axis) I_D_−V_GS_ curves of sensor. (Right axis) Corresponding measured leakage currents; (**e**) Drift trends of a single sensing site under electrolytes of pH 4, 7 and 10. Markers indicate the experimental data and lines the corresponding fitting (exponential) curves.

Upon O_2_ plasma, oxygen is introduced on the surface of Parylene C which is most likely attributed to the formation of chemical groups, such as carbonyl (-C=O-), carboxyl groups (-COOH) or hydroxyl groups (-OH), as demonstrated by previous studies [16,19]. Unlike Parylene C, which lacks binding sites, oxidized Parylene C is a chemically active surface able to capture H^+^ (Figure 3a), thus bringing the chemistry closer to the sensing site. Figure 3b depicts the transient response (output voltage of the dual operational amplifiers source-drain followers) of five sensing sites across three distinct electrolytes. The sensor exhibits a linear response within the pH range 4–10, indicating a chemical sensitivity of 23 mV/pH. A linear relationship between voltage and pH value is important to extrapolate straightforward correlations between detected voltage and H^+^ concentrations. As seen from Figure 3b, output voltage differs from channel to channel. This is primarily attributed to the fabrication process (*i.e.*, deposition of Parylene) which introduces non-uniformities over the entire set of sensing sites, attributing non-identical characteristics to the active transducers. Furthermore, mobile ions in the electrolytes under test interact with the membrane surface and affect its chemical composition and thickness especially after long-term use.

The minimum resolution of the sensor is 0.15 pH values and was determined through experiments within a pH range of 7–8, starting from a solution droplet of pH 7 and gradually increasing the pH of the droplet to determine the smallest pH change that the sensor is able to capture. The measured sensitivity may appear relatively low compared to Nernstian, yet the relevant differences between the various pH values are well distinguishable. The chemical sensitivity of the sensor depends on the oxygen plasma treatment, which in turn defines the degree of the induced hydrophilicity and the residual membrane thickness [20]. More profound oxidation of the Parylene membrane increases the number of free oxygen-based groups on the material's surface and thus the number of captured H^+^. Additionally, Parylene is significantly etched under plasma oxidation, yielding thinner sensing films on top of the electrode grid. Based on Coulomb's electrostatic law, thinner passivations account for an increased coupling of H^+^ [16], however, the small electrode size of the sensor limits the achievable pH sensitivity due to the limited number of protons impinging on the surface. Moreover, the substantially extended connection significantly increases the floating-gate capacitance resulting in a voltage-scaling, which justifies the observed sensitivity.

### 3.2. Hysteresis

Figure 3c shows the results of hysteresis measurements for the flexible sensor in the loop of pH = 10, 4, 7, 10, 7 and 4. Oxidized Parylene discloses a highly reactive behavior when it comes in contact with the solution for the first time, as indicated by the response of the sensor during the first three experiments in Figure 3c. The material's sensing capability appears to stabilize yet from the second experimental cycle. To evaluate the ability of the material to reproduce its response, hysteresis was calculated as the percentage that the sensor's (averaged) output voltage deviates from its initial response under the same electrolyte solution (Table 1). The sensor exhibits small hysteresis voltage shift values of 5 mV (pH 4), 12 mV (pH 7), whereas for pH 10 the hysteresis is larger (45 mV). It is worth mentioning that the oxidized Parylene C membrane tends to partially restore its induced hydrophilicity over time [20], which may also account for the observed deviations in the output response.

### 3.3. Electrical Functionality

The electrical functionality of the sensor was evaluated via an I_D_–V_GS_ sweep using a SCS-4200 semiconductor characterization system (Keithley, Cleveland, OH, USA). The MOSFETs gate voltage was swept from −1 to 0.5 V, while the drain and source voltages were fixed at 0.5 and 0 V respectively. The response of a single sensing site under electrolyte solutions of pH 4, 7 and 10 is depicted in Figure 3d. The right-axis of the figure demonstrates the corresponding measured leakage currents flowing through the Ag/AgCl reference electrode. Measurements at a constant current (100 μA), corresponding to the operational region of the MOSFETs, were used to assess the encapsulation integrity of the sensor (Table 1). Similarly to ISFETs, the gate-leakage current here is a straightforward indication of the encapsulation degradation. The leakage current in all cases remains at very low levels (1–14 nA), demonstrating the excellent encapsulation integrity of the sensor, which can be further enhanced by using thicker capping films and selectively oxidizing Parylene on top of the electrodes only, while preserving the isolation of the electrode tracks with a thick hydrophobic film [16].

### 3.4. Chemical Drift

The long-term stability of a sensor is particularly important, especially when the sensor needs to reliably operate inside an aqueous environment for a substantial amount of time. We evaluated the long-term stability of the sensor in terms of the voltage drift (ΔV). The main source of drift is the chemical interaction between the ions and the membrane, which causes alterations on the surface uniformity. The response of the flexible sensor was recorded for 1 h in a constant electrolyte environment (50 μL, pH 4, 7, 10) and average drift rates were extrapolated as the slope of the fitted lines (ΔV = A + Be^−Ct^) to the sensor's output excluding the first few minutes of transient response (Table 1). Figure 3e depicts representative drift trends of a single sensing site.

### 3.5. Local pH Detection

Local pH detection using the Parylene-based array was demonstrated through a diffusion experiment. Initially a microdroplet (50 μL) of pH 4 buffer solution was applied on the flexible platform.

**Figure 4.**
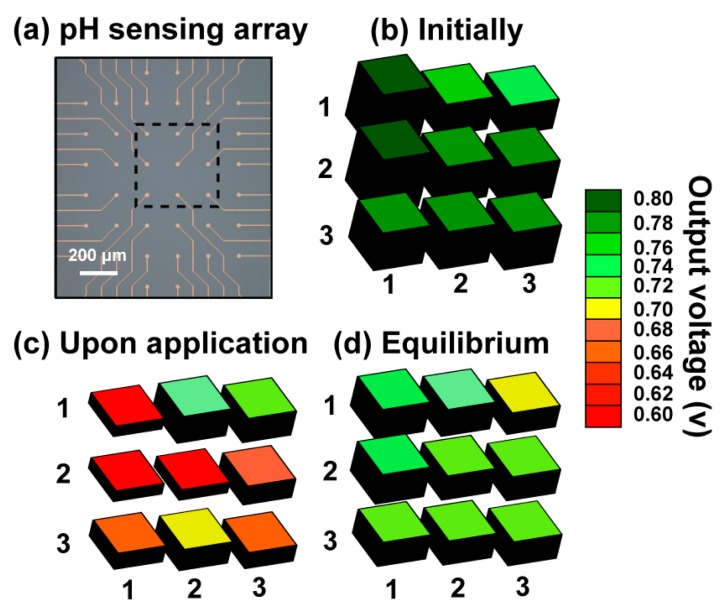
Spatiotemporal response of (**a**) the flexible array (3 × 3 region), as (**b**) solution of pH 4 is initially applied, (**c**) equally volumed solution of pH 7 is added and (**d**) equilibrium of ionic species is established (t = 5 s). Uncalibrated, unfiltered data.

A region consisting of a 3 × 3 electrode grid was continuously monitored, while an equal volume of pH 7 buffer solution was added. Figure 4 illustrates the response (uncalibrated data) of the sensing array throughout this experiment, as the solutions gradually mix and when the distinct ionic species within the two microdroplets are finally equally distributed.

## 4. Conclusions

In this paper, we have developed flexible Parylene-based high-density electrode arrays for functional monitoring of pH. Current applications for implantable devices based on Parylene C exploit the material either as an ion-blocking layer or as a flexible support substrate. Here, we present an integrated and multifunctional approach to use Parylene both as a flexible support medium and as an active pH-sensitive membrane within a single platform. The sensing arrays are microfabricated according to a simple sandwich structure of hydrophobic Parylene-metal-hydrophilic Parylene. The sensing platforms have been characterized via discrete MOSFETs. Experimental results indicate that the proposed platform can reliably map the local pH levels with a chemical sensitivity of 23 mV/pH, while it preserves a good encapsulation quality and small drift after a long-term use. Our approach can be exploited to establish hybrid flexible sensing platforms that will facilitate concurrent electrical and chemical recordings. This can yield a plethora of applications ranging from epidermal electronics (“bio-tattoos”) to implantable ultrathin sensing films for real-time physiological recordings.

## Figures and Tables

**Table 1.** Performance summary of the sensor.

**Buffer Solutions**	**Output Voltage ^a^ (V)**	**Hysteresis (%)**	**Leakage Current ^b^ (nA)**	**Drift Rate (mV/min)**
pH 4	0.76 ± 0.02	0.7	1.16	0.38
pH 7	0.68 ± 0.04	1.7	13.92	0.46
pH 10	0.62 ± 0.04	6.4	13.58	0.26

a Average measurements over 5 channels;

b Measurements at I_D_ = 100 μA.
